# Multi-view approach for the diagnosis of pulmonary hypertension using transthoracic echocardiography

**DOI:** 10.1007/s10554-017-1279-8

**Published:** 2017-12-11

**Authors:** Matthias Schneider, Anna Maria Pistritto, Christian Gerges, Mario Gerges, Christina Binder, Irene Lang, Gerald Maurer, Thomas Binder, Georg Goliasch

**Affiliations:** 1 Department of Internal Medicine II, Medical University of Vienna, Waehringer Guertel 18-20, 1090 Vienna, Austria; 2 Emergency Department, Division of Cardiology, San Paolo Hospital, Savona, Italy

**Keywords:** Pulmonary hypertension, Transthoracic echocardiography, TTE, Right heart catheterization, Peak tricuspid regurgitation velocity

## Abstract

Pulmonary hypertension (PH) is a disease with severe morbidity and mortality. Echocardiography plays an essential role in the screening of PH. The quality of the acquired continuous wave Doppler signal is the major limitation of the method and can greatly affect the accuracy of estimated pulmonary pressures. The aim of this study was to evaluate the clinical need to image from multiple ultrasound windows in patients with suspected pulmonary hypertension. We prospectively evaluated 65 patients (43% male, mean age 67.2 years) with echocardiography and right heart catheterization. 17% had invasively normal pulmonary pressures, 83% had pulmonary hypertension. Peak tricuspid regurgitation (TR) velocity was imaged in five echocardiographic views. Sufficient Doppler signal was recorded in 94% of the patients. Correlation for overall peak TR velocity with invasively measured systolic pulmonary artery pressure was r = 0.83 (p < 0.001). Considering all five imaging windows resulted in a sensitivity of 87%, and a specificity of 91% for correct diagnosis of PH with an AUC of 0.89, which was significantly better as compared to sole imaging from the right ventricular modified apical four-chamber view (AUC 0.85, p = 0.0395). Additional imaging from atypical views changed the overall peak TR velocity in 32% of the patients. A multiple-view approach changed the echocardiographic diagnosis of PH in 11% of the patients as opposed to sole imaging from an apical four-chamber view. This study comprehensively assessed the impact on clinical decision making when evaluating patients with an echocardiographic multiplane approach for suspected PH. This approach substantially increased sensitivity without a decrease in specificity.

**Electronic supplementary material:**

The online version of this article (10.1007/s10554-017-1279-8) contains supplementary material, which is available to authorized users.

## Background

Pulmonary hypertension is a disease with severe morbidity and mortality. Early detection is crucial to improve the outcome of these patients. However, until now misdiagnosis and late diagnosis are common [1, 2]. Echocardiography plays an essential role in the screening of pulmonary hypertension (PH).

By using the Bernoulli equation (ΔP = 4*v^2) the maximal velocity of the tricuspid regurgitant jet allows calculation of the pressure gradient between the right atrium and the right ventricle. By adding the estimated right atrial pressure this results in the right ventricular pressure which equals systolic pulmonary artery pressure in the absence of pulmonary stenosis. Because of common false assumptions regarding the right atrial pressure and therefore false calculations of systolic pulmonary artery pressure, the recent guidelines suggest using the maximal tricuspid regurgitant velocity for estimation of pulmonary pressures. Peak systolic tricuspid regurgitation velocity (TRvmax) correlates well with invasive measures of pulmonary pressure [3–5]. The guidelines suggest a cut-off of 3.5 m/s for high likelihood of the presence of PH. In the absence of other indicators of PH, an intermediate probability for the presence of PH is given if TRvmax is 2.9–3.4 m/s [6].

The angle of Doppler interrogation when measuring the maximal TR velocity can greatly affect the accuracy of estimated pulmonary pressures. The guidelines for the echocardiographic assessment of the right heart in adults therefore suggest imaging from several windows and using the signal with the highest velocity for calculations [7]. While the guidelines emphasize a multiple view approach, this is often not implemented in daily clinical practice and patients with PH are oftentimes diagnosed with late-stage disease [2]. However, early diagnosis is crucial for prognosis and the worse the World Health Organization (WHO) functional class at time of diagnosis the worse the survival chances [8–11]. So far there has not been a systematic evaluation of how a comprehensive assessment of all available imaging windows affects diagnostic accuracy. Furthermore, it is unclear how the results from the various windows differ and which view correlates best with invasively measured pressures. Therefore, the aim of this study was to comprehensively evaluate the clinical need to image from multiple ultrasound windows in patients with suspected pulmonary hypertension.

## Methods

### Study population

We prospectively included all adult patients with clinically indicated right heart catheterization (RHC) between July 2015 and July 2016. Transthoracic echocardiography was performed in all patients shortly before RHC. The study was conducted in accordance with the amended Declaration of Helsinki. The ethic committee of the Medical University of Vienna approved the conduct of the study (EK# 2012/2014). All patients gave written informed consent before enrollment. Exclusion criteria were patients < 18 years of age.

### Transthoracic echocardiography

Transthoracic echocardiograms (2D, Doppler) were performed with echocardiography systems equipped with 3.5 MHz transducers (Vivid E9, Vivid S70; General Electric Healthcare) according to the recommendations and guidelines by the American Society of Echocardiography and the European Association of Cardiovascular Imaging [7, 12].

In all patients peak CW Doppler velocity of the tricuspid regurgitation jet was systematically measured in the parasternal long-axis view of the RV inflow, the parasternal short axis view of the basal RV, the RV modified apical four chamber view, the apical long axis view of RV inflow, and the subcostal four chamber view (supplemental Fig. 1). For all velocities, the mean of three measurements was used for calculations. In patients with atrial fibrillation, the mean of six measurements was used for calculations. Any higher signal measured from one of the additional views was considered a change in overall peak TR. All echocardiograms were performed by an experienced physician. All measurements were performed offline by an experienced investigator. Intra-observer and inter-observer variability for TRvmax were assessed in ten randomly selected patients. Inter-observer agreement and intra-observer consistency were calculated by using interclass correlation coefficients (ICCs) and a 95% confidence interval (95% CI).

Probability for the presence of pulmonary hypertension was assessed according to the current guidelines. TRvmax of ≤ 2.8 m/s or a TR signal not measurable was considered as low probability, TRvmax 2.9–3.4 m/s was considered as intermediate probability, and TRvmax > 3.4 m/s was considered as high probability for the presence of pulmonary hypertension [6].

### Invasive hemodynamic measurement

Invasive hemodynamic assessment was performed in all study participants. Hemodynamic measurements were performed using a 7F Swan-Ganz catheter (Edwards Lifesciences GmbH, Austria) via a femoral access. Pressures were documented as average of eight measurements over eight consecutive heart cycles using CathCorLX (Siemens AG, Berlin and Munich, Germany). In addition to mean pulmonary arterial wedge pressure (mPAWPcath), the systolic (sPAPcath), diastolic (dPAPcath), and mean (mPAPcath) PA pressures were measured. In a subgroup of patients (n = 53) left ventricular end-diastolic pressure was measured via left heart catheterization where clinically indicated.

### Statistical analysis

Continuous variables are given as mean ± standard deviation (SD). Pearson correlation coefficients were calculated to compare invasive with echo measurements. Specificity and sensitivity were calculated for the different imaging views. Area under the curve (AUC) of the ROC curve was calculated to examine the power of the different measurements. A p value ≤ 0.05 was considered statistically significant. SPSS Version 24 (IBM SPSS, USA) was used for all analyses.

## Results

### Patient characteristics

Sixty-five patients fulfilled the study protocol with complete RHC and echocardiography data sets. Of these, 28 (43%) patients were male. The mean age was 67.2 years (range 19–89 years). All of the patients were admitted to the cardiology department for diagnostic work-up of suspected PH. None of the patients had to be excluded due to poor image quality.

### Invasive hemodynamic and echocardiographic measurements, classification of pulmonary hypertension

At RHC eleven (17%) patients had a mean pulmonary artery pressure (mPAPcath) of < 25 mmHg and were classified as non-PH. Fifty-four (83%) patients had an invasively measured mPAPcath of ≥ 25 mmHg and were classified as having PH. Eleven (17%) patients had mild pulmonary hypertension (mPAP 25–29 mmHg), and 43 (66%) patients had severe pulmonary hypertension (mPAP > 30 mmHg). Mean mPAWPcath was 13.6 ± 6.7 mmHg, mean sPAPcath was 61.0 ± 24.5 mmHg, and mean mPAPcath was 37.6 ± 14.9 mmHg. Left ventricular end-diastolic pressure was obtained in 53 patients (82%). Detailed echo characteristics are displayed in Table 1.

**Table 1:** Patient characteristics, echocardiographic and invasive data (n = 65)

Patient characteristics	
Age, mean years (range)	67.2 (19–89)
Male gender, n (%)	28 (43%)
Classification of pulmonary hypertension (PH)	
PH, n (% in relation to all patients with PH)	54 (100%)
Pulmonary arterial hypertension, n (%)	8 (15%)
PH due to left heart disease, n (%)	23 (43%)
PH due to pulmonary disease, n (%)	10 (18%)
CTEPH, n (%)	13 (24%)
Normal pulmonary pressures (mPAP < 25 mmHg)	
Number of patients, n (%)	11 (17%)
Tricuspid regurgitation ≥ moderate, n (%)	2 (3%)
Mild pulmonary hypertension (mPAP 25–29 mmHg)	
Number of patients, n (%)	11 (17%)
Tricuspid regurgitation ≥ moderate, n (%)	0 (0%)
Severe pulmonary hypertension (mPAP > 30 mmHg)	
Number of patients, n (%)	43 (66%)
Tricuspid regurgitation ≥ moderate, n (%)	21 (32%)
Echocardiographic data	
LVF ≥ moderately reduced, n (%)	9 (14%)
RVF ≥ moderately reduced, n (%)	22 (34%)
Aortic stenosis ≥ moderate, n (%)	8 (12%)
Aortic regurgitation ≥ moderate, n (%)	9 (14%)
Mitral stenosis ≥ moderate, n (%)	1 (2%)
Mitral regurgitation ≥ moderate, n (%)	22 (34%)
Tricuspid regurgitation ≥ moderate, n (%)	23 (35%)
Invasive data, right heart catheterization	
Mean PCWP, mmHg (± SD)	13.6 (± 6.7)
LVEDP, mmHg (± SD)	13.9 (± 6.9)
Systolic PA pressure, mmHg (± SD)	61.0 (± 24.5)
Mean PA pressure, mmHg (± SD)	37.6 (± 14.9)

Of the 54 patients with pulmonary hypertension, 15% had idiopathic pulmonary arterial hypertension, 43% had pulmonary hypertension due to left heart disease, 18% had pulmonary hypertension due to pulmonary disease, and 24% had chronic thromboembolic pulmonary hypertension (Table 1).

### Echocardiographic TR velocity assessment

In 4 (6%) patients no TR signal could be measured. Considering all five windows sufficient TR signal was recorded in 61 (94%) patients. A TR signal was available in 30 (46%) patients in the parasternal long-axis view of the RV inflow, in 29 (45%) patients in the parasternal short axis view of the basal RV, in 61 (94%) in the RV modified apical four chamber view, in 50 (77%) patients in the apical long axis view of RV inflow, and in 17 (26%) patients in the subcostal four chamber view (Table 2). In concordance with clinical practice, we considered a missing signal as estimated normal pulmonary pressure.

**Table 2:** Sensitivity, Specificity, area under the curve (AUC) of multiple echocardiographic views

	Maximal TR signal	Parasternal long-axis view	Parasternal short axis view	Apical four chamber view	Apical long axis view	Subcostal four chamber view
Signal available	94%	46%	45%	94%	77%	26%
Sensitivity	87%	32%	33%	80%	67%	22%
Specificity	91%	91%	100%	91%	100%	100%
AUC	0.89	0.61	0.67	0.85	0.83	0.61
95% CI	0.8–0.98	0.47–0.75	0.54–0.79	0.75–0.95	0.75–0.91	0.48–0.75

Compared with the other views peak TR velocity was highest in the parasternal long-axis view of the RV inflow in seven patients, in the parasternal short axis view of the basal RV in three patients, in the RV modified apical four chamber view in 20 patients, in the apical long axis view of RV inflow in 11 patients, and in the subcostal four chamber view in five patients. In 15 patients the same peak velocity was recorded from several angles. Compared to sole imaging from the RV modified apical four chamber view, additional imaging from atypical views resulted in higher overall peak TR velocity in 21 (32%) patients. The higher TR velocity did not change echocardiographic classification in 14 of the 21 patients. In four of the definite PH patients peak TR signal was below 2.9 m/s in the apical four chamber view but above 2.9 m/s in at least one other imaging window. In three of the PH patients peak TR signal was below 3.5 m/s in the apical four chamber view but above 3.5 m/s in at least one other imaging window. In none of the patients higher gradients from additional imaging windows resulted in an over-estimation of pulmonary hypertension. Since only higher but not lower velocities were accounted for in the additional views, there were no cases where a potential PH patient was downgraded via additional imaging.

Sufficient TR signal was available in 61 (94%) patients in the RV modified apical four chamber view. Sensitivity for correct classification of PH was 80%. Specificity was 91%. AUC was 0.85 (SD 0.06, 95% CI 0.75–0.95). Sufficient TR signal was available in 50 (77%) patients in the apical long axis view of RV inflow. Sensitivity for correct classification of PH was 66.7%. Specificity was 100%. AUC was 0.83 (SD 0.05, 95% CI 0.75–0.91). For sensitivity, specificity, and AUC of the remaining views see Table 2.

Considering all five imaging windows resulted in a sensitivity of 87%, and a specificity of 91% with an AUC of 0.89, which was significantly better as compared to sole imaging from the right ventricular modified apical four-chamber view (AUC 0.85, p = 0.0395). The multi-view approach resulted in a significantly better AUC compared to sole assessment of the parasternal long-axis view of the RV inflow (p < 0.01), the parasternal short axis view of the basal RV (p = 0.017), and the subcostal four chamber view (p < 0.01). TRvmax demonstrated good reproducibility with an interclass correlation coefficient for intra-observer variability of 0.99 (95% CI 0.976– 0.995) and for inter-observer variability of 0.969 (95% CI 0.0.931–0.985).

Bivariate correlation was calculated for maximal TR velocity of each of the imaging windows with invasively measured mean pulmonary artery pressure (mPAP). Correlation was statistically significant for each imaging window. The subcostal four chamber view showed the best correlation with r = 0.83 (p < 0.001). Both the RV modified apical four chamber view and the apical long axis view of RV inflow had the same correlation with r = 0.79 (p < 0.001). Correlation with the parasternal long axis view of the RV inflow was r = 0.51 (p < 0.001), correlation with the parasternal short axis view of the basal RV was r = 0.49 (p = 0.01). Overall peak TR velocity correlated with r = 0.78 (p < 0.001).

Correlation with invasively measured systolic pulmonary artery pressure (sPAPcath) was good as well. Correlation for overall peak TR velocity was r = 0.83 (p < 0.001), for values from the apical long axis view of RV inflow r = 0.82 (p < 0.001), for values from the subcostal four chamber view r = 0.81 (p < 0.001). Best correlation was achieved by the RV modified apical four chamber view (r = 0.85, p < 0.001). Measurements from the parasternal long axis view of the RV inflow correlated with r = 0.56 (p < 0.001), measurements from the parasternal short axis view of the basal RV with r = 0.62 (p < 0.001). For detailed correlations see Table 3.

**Table 3:** Correlation of peak TR signal with invasively measured mean PAP and PASP

	Image window	N	Correlation	p Value
Correlation of peak TR signal with invasively measured mean PAP	Maximal TR signal	61	0.78	< 0.001
	parasternal long-axis view of the RV inflow	30	0.51	< 0.001
	the parasternal short axis view of the basal RV	29	0.49	0.01
	RV modified apical four chamber view	61	0.79	< 0.001
	apical long axis view of RV inflow	50	0.79	< 0.001
	subcostal four chamber view	17	0.83	< 0.001
Correlation of peak TR signal with invasively measured PASP	Maximal TR signal	61	0.83	< 0.001
	parasternal long-axis view of the RV inflow	30	0.56	< 0.001
	the parasternal short axis view of the basal RV	29	0.62	< 0.001
	RV modified apical four chamber view	61	0.85	< 0.001
	apical long axis view of RV inflow	50	0.82	< 0.001
	subcostal four chamber view	17	0.81	< 0.001

## Discussion

This is the first study evaluating the impact on clinical decision making when systematically evaluating patients with an echocardiographic multiplane approach for suspected PH. According to our data atypical imaging changes the echocardiographic classification of PH in 11% of the patients as opposed to sole imaging from an apical four chamber view. Sensitivity increased substantially without a decrease in specificity.

Historical data reveals the poor prognosis of PH with a median survival of 6 months in untreated patients diagnosed with WHO functional class IV and a median survival of 6 years in untreated patients diagnosed with WHO functional class I and II [11]. The introduction of specific drugs for targeted therapy of PH has substantially decreased mortality and hospitalization rates. The rate of disease progression can be reduced and the burden of symptoms can be improved dramatically if diagnosis is made at an early stage [13]. Thus, it is crucial to make the definite diagnosis as early as possible. However, data from the French national registry suggests that PH is diagnosed in WHO functional class III or IV in 75% of the patients [2]. Thus, it remains a challenge for echocardiographers to make the diagnosis as early as possible. At the same time, the number of unnecessary RHCs has to be minimized in order to avoid possible complications of invasive imaging.

This study sought to reduce the number of false-negatives when screening patients with suspected PH. The beam-to-flow angle is essential when using CW Doppler to determine maximal tricuspid regurgitant jet velocity. Oftentimes eccentric TR jets lead to inconclusive CW Doppler envelopes and maximal TR velocity is underestimated in these patients. Therefore, it is important to consider atypical imaging windows in order to reduce the beam-to-flow angle as much as possible. To determine the clinical relevance of imaging from different angles we evaluated the change in clinical decision making through systematic imaging from different angles in opposition to only imaging from one standard view.

The most commonly used imaging window for measurement of peak TR velocity is the RV modified apical four chamber view. Compared to sole imaging from this view, additional imaging from atypical views resulted in higher overall peak TR velocity in 21 (32%) patients. In 7 (11%) patients, the documentation of atypical views changed not only the peak TR velocity but resulted in a different echocardiographic classification of PH and helped make the correct diagnosis.

Moderate and severe tricuspid regurgitation (TR) was predominantly present in patients with severe pulmonary hypertension. None of our patients with mild pulmonary hypertension had more than mild-to-moderate TR. In clinical practice it is oftentimes easier to detect an accurate TR jet velocity in patients with more pronounced TR. Nevertheless, in our patient collective the effect of imaging from multiple angles was present in mild PH as well as in severe PH. Of the seven patients where multiple plane imaging changed the echocardiographic classification of PH, three had mild PH and four had severe PH.

As presented in previous trials, our data confirmed good overall correlation of sPAPcath as well as mPAPcath with all acquired TR signals [3, 14]. However, previous trials demonstrated high accuracy but only moderate precision of absolute PASP values calculated from TRvmax signals despite good overall-correlations [4, 5]. Therefore, not a precise determination of PASP but the differentiation between PH patients and non-PH patients by using the TRvmax signal seems to be the primary goal of echocardiography in patients with suspected PH.

Sufficient TR signal was available in 94% of the patients in the RV modified apical four chamber view, and in 77% of the patients in the apical long axis view of RV inflow. Sensitivity for correct diagnosis of PH was 79.6 and 66.7% respectively, AUC for correct diagnosis was 0.85 and 0.83 respectively. The apical long axis view of RV inflow seems to be most important aside from the apical four chamber view. Sensitivity was low in the remaining three imaging windows, sufficient TR signal was available in < 50% of the cases in these views. However, if available, these signals correlated significantly with invasively measured PASP and mPAP. Combining all five measurements and using the maximal TR velocity lead to an increase in sensitivity to 87% without losing specificity (90.9%). The AUC increased to 0.89. According to these results, the RV modified apical four-chamber view was the single view with best sensitivity and with best availability. Especially in cases with poor signal quality additional views helped make the correct diagnosis.

Even though it is possible to include and exclude the presence of pulmonary hypertension with good sensitivities and specificities by the multiple view approach, it is important to say that it is still not possible to make assumptions about the etiology of pulmonary hypertension by echocardiography. This remains an important goal for further studies.

This study has limitations. Echocardiography and RHC were not performed simultaneously in all of the patients. A time difference between the echocardiographic examination and the RHC can influence the measured pressures. Echo was performed shortly prior to catheterization in order to minimize this potential source of bias. Mean time interval between echocardiography and catheterization was less than 1 day. Another potential limitation is that our data reflects the experience of a single tertiary care center. However, the potential advantages of a single-center approach are the enrolment of a homogenous patient population, the adherence to a consistent clinical routine, and a consistent quality of imaging procedures and RHC. The study population was small with only 65 patients. Results showed statistical significance nevertheless and should persuade clinicians to implement systematic measurements into their daily routine.

## Conclusion

Patients with PH can easily be misdiagnosed if imaging is not performed thoroughly. The presented standardized systematic echocardiographic approach reaches excellent sensitivity and specificity with non-invasive imaging. Patients with suspected PH have to be imaged systematically from different angles to minimize highly consequential false negatives as well as false positives.

## Electronic supplementary material

Below is the link to the electronic supplementary material.

Supplemental figure 1: Panel A: Parasternal long-axis view of the RV inflow. Panel B: Parasternal short axis view of the basal RV. Panel C: RV modified apical four chamber view. Panel D: Apical long axis view of RV inflow. Panel E: Subcostal four chamber view. (JPG 308 KB)
