# Supplementary figures and images for: Multi-view approach for the diagnosis of pulmonary hypertension using transthoracic echocardiography
Source: Int J Cardiovasc Imaging. 2017 Dec 11;34(5):695–700. doi: 10.1007/s10554-017-1279-8 (PMC5889411; doi:10.1007/s10554-017-1279-8)

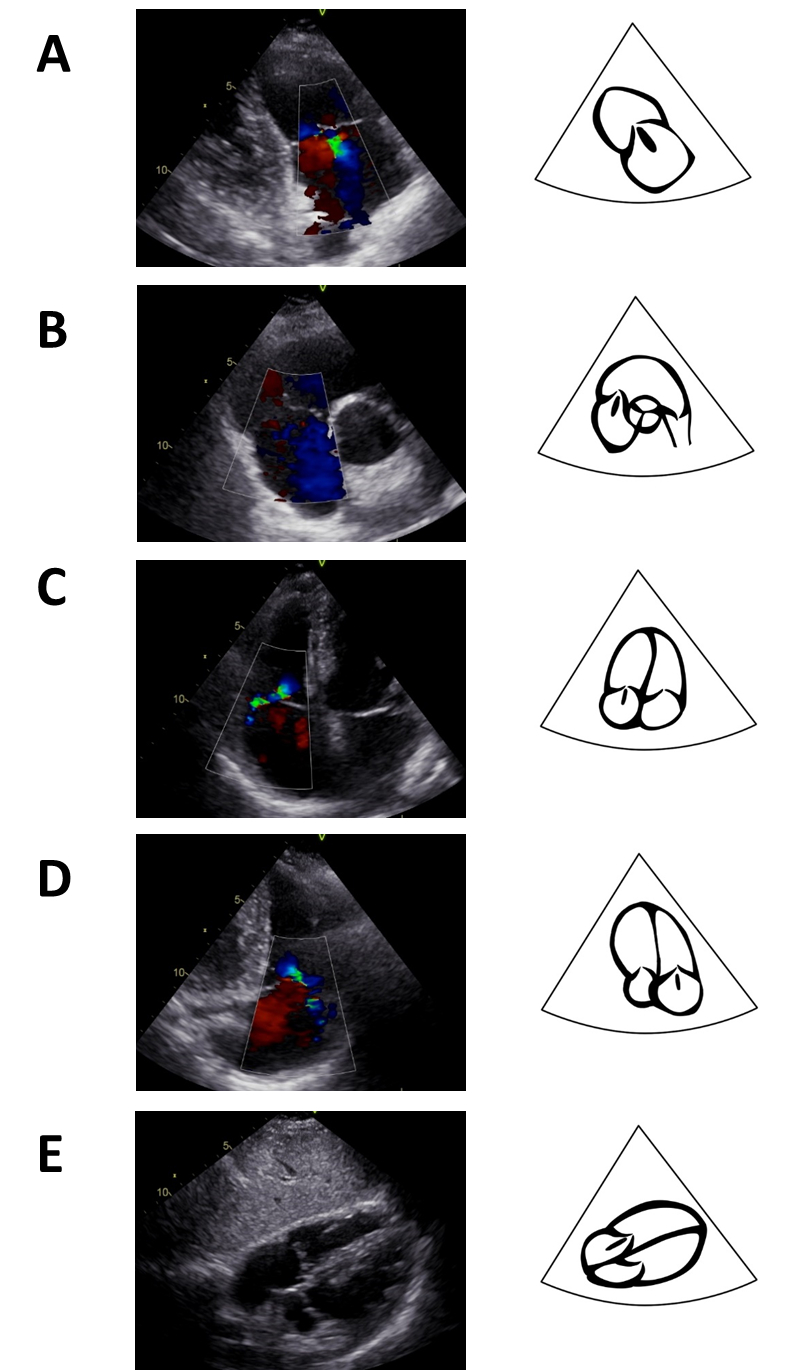

Supplement: Supplementary file 1 — Supplemental figure 1: Panel A: Parasternal long-axis view of the RV inflow. Panel B: Parasternal short axis view of the basal RV. Panel C: RV modified apical four chamber view. Panel D: Apical long axis view of RV inflow. Panel E: Subcostal four chamber view. (JPG 308 KB) [file 10554_2017_1279_MOESM1_ESM.jpg]
